# Criteria to Assess a Randomized Controlled Pilot Study: Response to Letter

**DOI:** 10.5812/hepatmon.10867

**Published:** 2013-04-17

**Authors:** Sonia Oveisi

**Affiliations:** 1 Metabolic Diseases Research Center, Qazvin University of Medical Sciences, Qazvin, IR Iran

**Keywords:** Randomized Controlled Trial, Non-Alcoholic Fatty Liver Disease

*Dear Editor,*

As some problems have been mentioned in article "Should We Rely on the Findings of Each Published Randomized Controlled Study?" (1), this letter has been provided to clarify them for journal's readerships.

Nowadays, Randomized Clinical Trial studies have been known as gold standard studies on condition that researchers pay attention on the most important factors in design such as:

1. Maximize statistical power in equal group sizes especially in subgroup analyses.
2. Minimize selection bias when randomization procedure will be unpredictable so that researchers cannot guess the next subject's group assignment based on prior treatment assignments. Also it is noticeable that balancing both known and unknown prognostic factors in the assignment of treatments is basic to approve it.
3. Minimize allocation bias when the treatment effect is confounded with the effect of the covariates.
4. Rectify statistical error of RCTs that contains both type I ("false positive") and type II ("false negative") (2-5).

It should be noted there are some other points in these studies that are in the second priorities, which can improve results, but it is conventional like biostatistics procedures. Many consider T test, ANOVA, and Chi Square test were developed over more than 60 years ago and the statistical methodology has been advanced. While these tests may still be appropriate, it is likely that additional statistical analysis will be considered as required analysis by many peer reviewers of journals (6). If we are interested in measuring dependent variables at the beginning and the end of the study at least twice and to compare treatment groups, not just looking at whether there has been any time dependent change in each group of subjects, we would have a multivariate repeated measurement especially when experimental hypotheses are stated in main topic rather than simply main effects or interactions. This statistical analysis is able to determine that there is a change in the variables levels of subjects over time (time effect) within-subjects, treatment effect between-subjects and the effect of time the same for both treatments. Therefore, with regarding to these advantages, authors are keen on using repeated measures in this study. Sometimes investigators detect effects in their experiment that were not expected or decide to explain almost any pattern of means, it would be appropriate to analyze and evaluate that pattern as multiple tests post-hoc. However many journals have recommended that mention of post-hoc comparisons should be avoided in the text (7). Otherwise, it is obvious that researchers have done many statistical analyses such as determining normal distribution of variables by valid methods or establishing the agreement between observers and intra-observer but it is not essential to report all details providing that journals are captivated to express the specific information. It should be noticed this trial study like the others is registered in the clinical trial registry (IRCT), although the journal does not have any tendency to report it. In addition, it should be pointed out that cut-off definition of some variables such as abnormal FBS or liver function test is health based and is associated with progress of metabolic syndrome components neither calculate on population percentiles. Despite authors have paid careful attention to write results, there are some errors. We did not have any missing data and Table below shows correct numbers and percentages. Finally authors appreciate everyone who pays attention to our randomized clinical pilot study and offers the more attention in design of these studies which can lead to achieve the more effective results as we have concerned in this study.

**Table.** Clinical and Non-Clinical Changes of Some Parameters in Three Groups of Intervention

	HOMA-IR		Cholesterol	
	Pre No. (%)	Post No. (%)	Pre No. (%)	Post No. (%)
**Pioglitazone**				
Clinical	12 (54.55)	5 (22.73)	11 (50)	7 (31/82)
Non Clinical	10 (45.45)	17 (77.27)	11 (50)	15 (68.18)
**Metformin**				
Clinical	13 (59.1)	8 (36.36)	9 (40.91)	6 (27.27)
Non Clinical	9 (4. 9)	14 (63.64)	13 (59.09)	16 (72.73)
**Silymarin**				
Clinical	13 (59.1)	13 (59.1)	10 (45.45)	7 (31.8)
Non Clinical	9 (40.9)	9 (40.9)	12 (54.55)	5 (68.2)
**Total**				
Clinical	38 (57.6)	26 (39.4)	30 (45.46)	20 (30.3)
Non Clinical	28 (42.4)	40 (60.6)	36 (54.55)	46 (69.7)

## References

[1] Kabir A (2013). Should We Rely on the Findings of Each Published Randomized Controlled Study?. Hepat Mon..

[2] Avins AL (1998). Can unequal be more fair? Ethics, subject allocation, and randomised clinical trials.. J Med Ethics..

[3] Buyse ME (1989). Analysis of clinical trial outcomes: some comments on subgroup analyses.. Control Clin Trials..

[4] Lachin JM (1988). Statistical properties of randomization in clinical trials.. Control Clin Trials..

[5] Rosenberger J (2012). STAT 503 - Design of Experiments..

[6] (2012). Guide for authors ,Anesthesia and analgesia the gold standard in Anesthesiology.. http://www.anesthesiaanalgesia.org/site/documents/ANEkit.pdf.

[7] Mills JL (1993). Data torturing.. N Engl J Med..

